# Recurrent Isolated Uvular Angioedema Associated With Intranasal Cocaine Use: A Case Report

**DOI:** 10.7759/cureus.56818

**Published:** 2024-03-24

**Authors:** Erika Tsutsui, Christian Gomez-Hernandez, Destiny Nguyen, Yuhong Yang, Songhui Ma

**Affiliations:** 1 Internal Medicine, Icahn School of Medicine at Mount Sinai, New York, USA; 2 Allergy and Immunology, Icahn School of Medicine at Mount Sinai, New York, USA

**Keywords:** angioedema, quincke’s disease, quincke's edema, isolated uvular angioedema, cocaine

## Abstract

Isolated uvular angioedema, or Quincke's disease, is a rare manifestation with various potential causes. This article presents the first documented case of recurrent isolated uvular angioedema associated with intranasal cocaine use. The patient, a 43-year-old man, exhibited acute symptoms of sore throat, throat swelling, and difficulty breathing, with a history of a similar episode a few years prior. Both episodes occurred following intranasal cocaine use. Examination revealed an enlarged uvula obstructing the airway. The patient was treated with epinephrine, antihistamines, and corticosteroids with resolution of the uvular edema. This case highlights the importance of considering cocaine as a potential causative agent in isolated uvular angioedema and emphasizes the need for patient education to avoid further cocaine use.

## Introduction

Isolated uvular angioedema, also known as Quincke's disease, is a rare but potentially serious condition characterized by localized swelling of the uvula, often triggered by various etiologies. Among these triggers, cocaine use has emerged as an important but less recognized cause, highlighting the diverse spectrum of factors that can lead to this condition. 

First described by Heinrich Quincke in 1882, Quincke's disease involves increased vascular permeability in the submucosal layer of the uvula, resulting in fluid extravasation and pronounced swelling. While allergic reactions, medications such as nonsteroidal anti-inflammatory drugs (NSAIDs) and angiotensin-converting enzyme (ACE) inhibitors, infections, and trauma are well-established triggers, the association with cocaine use underscores the need for vigilance in identifying less conventional etiologies. 

Patients with isolated uvular angioedema typically present with symptoms such as a sore throat, odynophagia (painful swallowing), dysphagia (difficulty swallowing), gagging, and hoarseness. In cases where cocaine use is implicated, a detailed history of substance use is crucial for accurate diagnosis and management. 

The rapid onset and potential for severe swelling in Quincke's disease necessitate prompt recognition and intervention to prevent airway compromise and obstruction. Differential diagnosis should include other causes of angioedema, including hereditary and acquired forms, to guide appropriate treatment strategies. 

In this case report, we present a case of isolated uvular angioedema triggered by cocaine use in a 43-year-old male patient with a history of substance abuse, highlighting the clinical presentation, diagnostic challenges, management approaches, and outcomes. By exploring this unique trigger, we aim to raise awareness of the diverse etiologies of Quincke's disease and emphasize the importance of a comprehensive patient evaluation for optimal care.

## Case presentation

A 43-year-old male presented to the Emergency Department (ED) complaining of acute onset sore throat, throat swelling, and difficulty breathing. Symptoms started while he was at a bar the previous night, prompting him to call for an ambulance. The patient denied consuming any food within six hours prior to symptom onset, having only consumed several glasses of beer, which had never caused issues in the past. He had experienced flu-like symptoms over the past week, including cough, rhinorrhea, and nasal congestion, and his daughter had recently been diagnosed with respiratory syncytial virus (RSV). He denied using any medications, including ACE inhibitors or NSAIDs, and had no family history of angioedema or recent insect bites. However, he admitted to intranasal cocaine use two hours before the symptom onset. 

Notably, the patient had a previous episode of acute uvular edema with difficulty breathing, leading to admission to the intensive care unit four years prior. Edema developed following cocaine use. He denied other triggers of edema, including allergy or infection.

**Figure 1:**
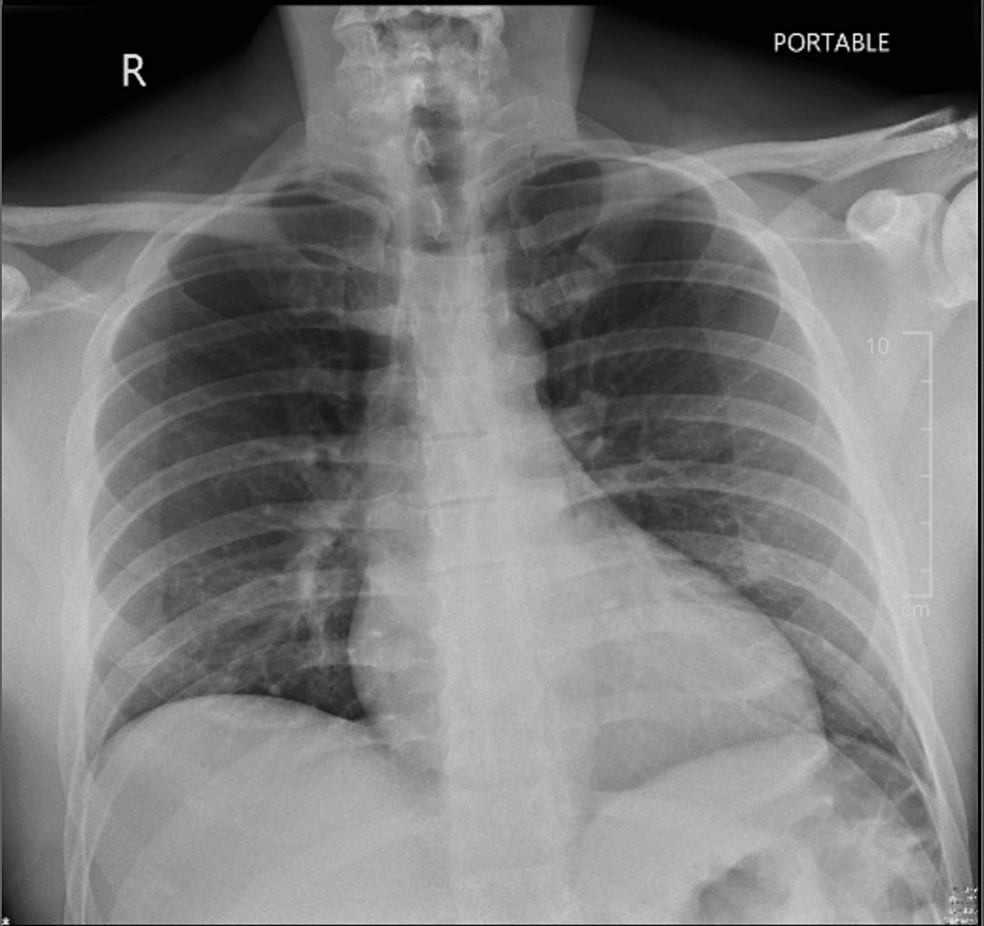
Chest X-ray on admission

In the ED, the patient was in respiratory distress in a tripod position. He was afebrile (98.4 °F), normotensive (128/81 mmHg), tachycardic (heart rate: 128 bpm), and tachypneic (respiratory rate: 17 breaths per minute) with oxygen saturation at 97% on room air. Physical examination revealed an enlarged uvula obstructing the airway, along with a hoarse voice. There was no edema of the tongue, lips, or face. There was no urticaria or rash. Cardiac and pulmonary auscultation findings were unremarkable. Blood work (Table 1) and infectious workup (Table 2) were unremarkable, and a chest X-ray (Figure 1) showed no signs of pneumonia or pulmonary edema. 

**Table 1:** Blood work BUN: blood urea nitrogen, EGFR: estimated glomerular filtration rate, CRP: C-reactive protein, ESR: erythrocyte sedimentation rate

	Test result	Reference rage
Hemoglobin (g/dL)	13.2	13.9-16.3
White blood cell (K/uL)	7.9	4.5-11.0
Neutrophils (K/uL)	6.0	1.9-8.0
Lymphocytes (K/uL)	1.4	1.0-4.5
Monopiles (K/uL)	0.3	0.2-1.0
Eosinophils (K/uL)	0.0	0.0-0.6
Basophils (K/uL)	0.1	0.0-0.2
Platelets (K/uL)	276	150-450
BUN (mg/dL)	18	6-23
Creatinine (mg/dL)	1.44	0.70-1.30
Sodium (mEq/L)	135	135-145
Potassium (mEq/L)	4.4	3.5-5.2
Chloride (mEq/L)	102	96-108
CO₂ (mEq/L)	19.1	22.0-30.0
Anion gap (mEq/L)	13.90	7-16
Calcium (mg/dL)	9.4	8.5-10.5
EGFR (mL/min/1.73m2)	62	>59
CRP (mg/L)	1.2	<5.1
ESR (mm/hr)	16	0-13
Total IgE (IU/mL)	32	6-495
Complement C4 (mg/dL)	30	10-40

**Table 2:** Infectious workup PCR: polymerase chain reaction, RS virus: respiratory syncytial virus, COVID-19: coronavirus disease 2019, EBV: Epstein-Barr virus

	Test result	Reference range
Adenovirus PCR	Not detected	
Metapneumovirus PCR	Not detected	
Parainfluenza virus PCR	Not detected	
RS virus PCR	Not detected	
Influenza virus PCR	Not detected	
Rhinovirus PCR	Not detected	
Enterovirus PCR	Not detected	
*Chlamydophila pneumoniae* PCR	Not detected	
*Bordetella pertussis* PCR	Not detected	
Mycoplasma PCR	Not detected	
Seasonal coronavirus PCR	Not detected	
COVD-19 PCR	Not detected	
*Cytomegalovirus* PCR	Not detected	
Mono spot	Not detected	
EBV capsid IgG (U/mL)	98.7	0.0-17.9
EBV capsid IgM (U/mL)	<36	0.0-35.9
Rapid group A *Streptococcus* PCR	Not detected	

The patient received immediate intramuscular epinephrine (0.3 mg, administered twice), along with intravenous antihistamines (diphenhydramine 50 mg and famotidine 20 mg), intravenous methylprednisolone (Solu-Medrol 125 mg), fresh frozen plasma (FFP) two units (as indicated in hereditary angioedema), and racemic epinephrine. A bronchoscopy through the nasal airway was performed an hour after epinephrine administration by the critical care consult team to assess the need for intubation, which revealed a normal epiglottis and vocal cords without significant swelling. The patient was monitored in the ED for six hours and then transferred to the general medicine floor. The uvular edema gradually improved with continued intravenous steroid and antihistamine, and on day 3 of admission, the patient was stable for discharge. He was provided with a two-day supply of oral prednisone (40 mg) to complete a five-day course of steroids, along with daily antihistamines. He was counseled that cocaine may have triggered both episodes, advised to avoid further cocaine use, and recommended to follow up with an allergist.

## Discussion

Isolated uvular angioedema presents a diagnostic challenge due to its rarity and diverse etiologies. Angioedema triggers include type 1 hypersensitivity reactions and other drug reactions, hereditary angioedema, and irritants [1]. 

In our case, isolated uvular angioedema developed in the setting of cocaine use on multiple occasions, supporting the role of cocaine as the likely trigger. Literature reports cases of angioedema due to the irritant effects of inhaled cannabis and cocaine [2-6].

The differential diagnosis also includes the following: Uvulitis may be associated with infection and viral infection may have been a cofactor. Although our patient had had flu-like symptoms the week before and his daughter had been ill with RSV, the viral studies were negative. There was also no evidence of bacterial extension from adjacent infections, such as Group A *Streptococcus* tonsilitis or pharyngitis. The patient was afebrile with no leukocytosis. 

Type 1 hypersensitivity reactions are a well-established cause of angioedema. Mast cell degranulation and histamine release contribute to the sudden onset of swelling [1]. However, our patient had no history of food or drug allergies. He denied eating six hours prior to the onset of symptoms and denied the use of any new medications or supplements. 

Drug-induced angioedema may also occur via non-IgE-mediated mechanisms. NSAIDs and ACE inhibitors are common culprits [1,7,8]. Our patient denied using these medications. 

Hereditary angioedema is a rare genetic disorder but should be considered. Patients have deficiency or dysfunction of C1 inhibitor, resulting in recurrent episodes of angioedema [9]. Our patient had normal C4 levels and no family history of angioedema.

## Conclusions

We report recurrent episodes of a rare type of angioedema in the setting of cocaine use. This case highlights the multifaceted nature of isolated uvular angioedema and the potential role of cocaine in its development. A comprehensive understanding of the various etiologies, including irritants, hypersensitivity reactions, drug-induced causes, and hereditary factors, is crucial for accurate diagnosis and appropriate management. Patients presenting with uvular angioedema should be assessed for recreational drug use and educated about the risks associated with cocaine to prevent further episodes. Healthcare providers should remain vigilant in exploring unusual triggers for angioedema, particularly in cases with atypical presentations.

## References

[1] Kaplan AP (2008). Angioedema. World Allergy Organ J.

[2] Gonçalves FM, Costa M, Campos AL, Cotter J (2022). Quincke's disease presenting after cocaine exposure. Cureus.

[3] Heard M, McMahon K, Nappe T (2020). A case of cocaine-induced uvular edema. Cureus.

[4] Hidalgo Mora JJ, Giménez de Llano E, Sempere Verdú E, Feliú Sagalá M (2002). Uvular angioedema after intranasal cocaine consumption [Article in Spanish]. Med Clin (Barc).

[5] Kestler A, Keyes L (2003). Images in clinical medicine. Uvular angioedema (Quincke's disease). N Engl J Med.

[6] Mohammadi L, Miller A, Ashurst JV (2016). Quincke's disease. J Family Med Prim Care.

[7] Varghese RT, McCann R, Khasawneh K, Jacob DG (2019). Uvular angioedema in a patient on angiotensinreceptorblocker. Postgrad Med J.

[8] Kuo D, Barish R (1995). Isolated uvular angioedema associated with ACE inhibitor use. J Emerg Med.

[9] Zuraw BL (2008). Clinical practice. Hereditary angioedema. N Engl J Med.

